# Clinical efficacy of Teriparatide combining Zhuanggu Zhitong capsules in the treatment of osteoporosis

**DOI:** 10.12669/pjms.41.11.12461

**Published:** 2025-11

**Authors:** Meng Cao, Panxiang Li, Shike Wu, Xiaoming Wu, Lan Yang

**Affiliations:** 1 Meng Cao Science and Education, Baoding No.1 Central Hospital, Baoding, 071000 Hebei, China; 2 Panxiang Li Department of Orthopedics, Affiliated Hospital of Hebei University, Baoding, 071000 Hebei, China; 3 Shike Wu Department of Orthopedics, Affiliated Hospital of Hebei University, Baoding, 071000 Hebei, China; 4 Xiaoming Wu Department of Orthopedics, Affiliated Hospital of Hebei University, Baoding, 071000 Hebei, China; 5 Lan Yang Department of Nutritional, Affiliated Hospital of Hebei University, Baoding, 071000 Hebei, China

**Keywords:** Bone mineral density, Bone metabolism indicators, Dysfunction, Life quality, Osteoporosis, Pain, Teriparatide, Zhuanggu Zhitong Capsules

## Abstract

**Objective:**

To investigate the clinical efficacy of Teriparatide(TPTD) combining Zhuanggu Zhitong Capsules(ZZC) in the treatment of primary osteoporosis.

**Methodology:**

This single-center, retrospective cohort study consecutively enrolled eighty patients with primary osteoporosis who received treatment from August 2020 to August 2022. Patients were divided into two groups based on the treatment they received: the control group (n=40) and the treatment group (n=40). Both therapies continued for six months. Bone density, bone metabolism indicators, Visual Analogue Scale (VAS) score, Oswestry Disability Index (ODI) score, and Short Form Health Survey-36 (SF-36) score were compared between the two groups before and after treatment. The total effective rate and adverse reactions were also calculated.

**Results:**

After treatment, the treatment group showed significantly lower levels of bone alkaline phosphatase (BALP), β-carboxy-terminal telopeptide of Type-I collagen (β-CTX), VAS score, and ODI score compared to baseline (all P<0.05), with greater reductions than the control group (P<0.05). Total hip bone mineral density (BMD) T-values, lumbar L2-L4 BMD T-values, bone GLA protein (BGP) levels, and SF-36 scores were significantly higher in both groups post-treatment (P<0.05), with greater improvements in the treatment group (P<0.05). The total efficacy of the treatment group (90.0%) was significantly higher than that of the control group (67.5%, P<0.05). No serious adverse reactions occurred, and the incidence of mild adverse reactions (nausea, dizziness, abdominal distension) was 5.0% (2/40) in the treatment group and 10.0% (4/40) in the control group (P>0.05).

**Conclusions:**

The combination of Teriparatide and Zhuanggu Zhitong Capsules is highly effective in treating primary osteoporosis, demonstrating rapid symptom relief, regulation of bone metabolism, and improvement in functional impairment and quality of life.

## INTRODUCTION

Osteoporosis is a systemic bone disease characterized by reduced bone mass, microarchitectural deterioration of bone tissue, and increased fracture risk.^1^ It significantly impairs patients’ ability to live independently and imposes a heavy economic burden on healthcare systems.^2^ In traditional Chinese medicine (TCM), osteoporosis is often attributed to “kidney deficiency and blood stasis,” and TCM therapies (e.g., Chinese patent medicines) are considered advantageous due to their lower cost, fewer adverse reactions, and simpler administration compared to decoctions.^3^ Zhuanggu Zhitong Capsules(ZZC), composed of six herbs (Psoralea Fruit, Epimedii Herba, Glossy Privet Fruit, Wolfberry Fruit, Drynariae Rhizoma, and Cibotii Rhizoma), exert effects of tonifying the kidneys, strengthening bones, and relieving pain. However, high-quality clinical evidence regarding the combination of Teriparatide(TPTD) with ZZC remains limited. Therefore, this retrospective study aims to evaluate the effectiveness and safety of this combination therapy compared to TPTD alone in patients with primary osteoporosis, focusing on bone mineral density (BMD), bone metabolism markers, pain relief, functional improvement, and quality of life.

## METHODOLOGY

This was a single-center, retrospective cohort study. Data were retrospectively extracted from the electronic medical record (EMR) system of Baoding No.1 Central Hospital. A consecutive sampling method was used to include eighty patients with primary osteoporosis who received treatment between August 2020 and August 2022.

Patients were divided into two groups based on the treatment regimen they received: the control group (Teriparatide alone, n=40) and the treatment group (Teriparatide + Zhuanggu Zhitong Capsules, n=40). As a retrospective study, no randomization or blinding procedures were performed as the treatment regimens were determined clinically prior to the initiation of this data analysis. The treatment group was formed by 23 male and 17 females patients 50~75 years old (mean age: 65.22±3.41) with the course of disease ranging from 2-14 years [mean: (7.55±2.15)]. Concerning 25 male and 15 female cases in the control group, they are 52~73 years old (mean age: 65.25±3.40) with courses of disease lasting for 2~13 years [mean: (7.53±2.17) years], No statistical matching was performed between the groups.There is no statistically significant difference in general data between the two groups (*P*>0.05).

### Ethics approval:

This retrospective analysis of anonymized clinical data was approved by the Institutional Ethics Committee of Baoding No.1 Central Hospital (No.:HDFYLL-KY-2023-136; approval date: 06 July, 2023). The need for individual informed consent for this specific analysis was waived by the committee due to the retrospective nature of the study. All patients had provided written informed consent for their original treatments.

### Diagnostic criteria:

Western diagnosis observes the following stipulations of “Primary Osteoporosis” in the Guidelines on Primary Osteoporosis Diagnosis and Treatment (2022).^4^ With respect to diagnosing the syndrome of Chinese medicine, referred to the Guidelines on Clinical Research of New Traditional Chinese Medicine^5^ and the Expert Consensus on Primary Osteoporosis Prevention and Treatment by Traditional Chinese Medicine (2020).^6^ Syndrome differentiation proves that it is kidney deficiency and blood stasis. Primary symptoms cover sharp pain in the lower back, fixed and unpalpable pain, and soreness and weakness of the waist and knees; and muscle contracture, impotence of the lower limb, difficulties in walking, tongue nature being pale purple are all its secondary symptoms.

### Inclusion criteria:

- Aged 50~75 years.
- Diagnosed with primary osteoporosis (Western and TCM criteria, detailed below).
- Provided written informed consent
- Agreed to complete 6-month follow-up.

### Exclusion criteria:

- Secondary osteoporosis (e.g., due to hyperparathyroidism, glucocorticoid use).
- Severe organ dysfunction (liver/kidney)
- Malignant tumors
- Metabolic bone diseases (e.g., multiple myeloma)
- Severe spinal deformity or osteoarthritis
- Prior joint replacement
- Allergy to Teriparatide or Zhuanggu Zhitong Capsules.

### Therapeutic methods:

All patients took Vitamin-D and calcium and received healthy lifestyle guidance (e.g., Quitting smoking, drinking, balanced nutrition, and regular exercise) and psychological adjustment interventions, etc. While subcutaneous injection of Teriparatide Injection (Lilly France; SFDA approval number: S20170032; specification: 20 μg:80 μl) was performed at a dose of 20 μg once a day in the control group. For the treatment group, Teriparatide was combined with Zhuanggu Zhitong Capsule (Sichuan Medco Pharmaceutical Stock Co., Ltd.; SFDA approval number: Z20050118; specification: 0.45g) at a dose of four capsules per time and three times a day. Treatment for both groups continued for six months. Patient compliance was assessed by reviewing medication administration records and pharmacy dispensing records within the EMR system.

### Observation targets and methods:

- *Bone density:* Using a dual energy X-ray bone density analyzer to detect the overall t-value of L2-L4 bone density in the left hip and lumbar spine.
- *Bone metabolism indicators:* Before and after treatment, venous blood was collected from patients to test levels of bone alkaline phosphatase (BALP), bone gla protein (BGP) and carboxy-terminal cross-linking telopeptide of type I collagen (β-CTX) by the double-antibody sandwich enzyme-linked immunosorbent assay (ELISA).
- *Pain scores:* Visual analogue scales (VAS) were selected to evaluate pain intensity of patients before/after treatment; the intensity was classified into 0~10 scales. While 0~3 represents mild pain, 4~7 and 8~10 respectively refer to moderate and severe pain.
- *Oswestry disability index (ODI) based scores:* This questionnaire covered 10 questions in total, and 0~5 point(s) could be assigned to each question. The higher the score assigned to a question is, the more severe the corresponding dysfunction will be. No dysfunction was denoted by 0~4, and mild dysfunction by 5~14, moderate dysfunction by 15~24, severe dysfunction by 25~34, and extremely severe dysfunction by over 34 points.
- *Life quality scores:* The SF-36 questionnaire was adopted to assess patients’ life quality before and after treatment, totaling 100 points. The higher a score is, the better patients’ life quality will be.
- *Clinical efficacy*.^7^ Being significantly effective means that uncomfortable symptoms such as pain are completely relieved after treatment and the bone mineral density is also substantially improved. Regarding being effective, it signifies that uncomfortable symptoms such as pain are obviously relieved after treatment and the bone mineral density shows no decrease. For ineffective cases, uncomfortable symptoms such as pain are not relieved at all after treatment; and their bone mineral density shows no improvements or even drops. Overall response rate = Values of being significantly effective + Values of being effective.
- *Safety:* Statistics about adverse reactions in both groups were made, including nausea, dizziness and abdominal distension.

Adherence was assessed via monthly phone calls and review of medication dispensing records. Compliance was defined as taking ≥80% of prescribed doses.

### Statistical processing:

Data were analyzed using SPSS24.0. Relevant measurement data are denoted as Mean ± Standard Deviation (*χ̅*±*S*). While pairwise samples t-test is selected for intra-group comparison, independent samples t-test is performed for inter-group comparison. Enumeration data are represented by frequency and %, in which case, inter-group comparison of data is carried out by means of the chi-squared test. If *P*<0.05, the differences are considered statistically significant.

## RESULTS

Baseline characteristics of the two groups are shown in Table-I. No significant differences were observed in age, gender, or disease duration (P>0.05). Table-II indicates that t-values of bone mineral density in the left hip and lumbar L2-L4 after treatment are obviously higher than those before treatment of both groups(*P*<0.05), and the treatment group was higher than the control group (P<0.05).

**Table-I:** Baseline characteristics of the two groups.

Variable	Control Group (n=40)	Treatment Group (n=40)	P-value
Age (years)	65.25±3.40	65.22±3.41	0.97
Gender (male/female)	25/15	23/17	0.65
Disease duration (years)	7.53±2.17	7.55±2.15	0.96

**Table-II:** Bone mineral density comparison before/after treatment in observation and control groups (*χ̅*±*S*) Unit: g/cm^2^.

Items	Treatment group (n=40)		Control group (n=40)	
	Before treatment	After treatment	Before treatment	After treatment
Lumbar L2-L4	-3.05±0.54	-2.55±0.45*^△^	-3.09±0.57	-2.87±0.43*
Left hip	-2.71±0.51	-2.40±0.46*^△^	-2.74±0.53	-2.55±0.49*

***Note:*** Compared with values before treatment of the same group,

* *P<*0.05; comparison with the control group after treatment,

△ *P<*0.05.

It can be observed from Table-III that BALP and β-CTX levels in both groups after treatment are lower than those before treatment (P<0.05), and the observation group was lower than the control group (P<0.05); after treatment, the BGP levels in both groups were higher than before treatment (P<0.05), and the observation group was higher than the control group (P<0.05).

**Table-III:** Comparison of variations in bone metabolism indicators before/after treatment in observation and control groups (*χ̅*±*S*) Unit: μg/L.

Items	Treatment group (n=40)		Control group (n=40)	
	Before treatment	After treatment	Before treatment	After treatment
BALP	17.08±1.73	9.11±0.91*^△^	17.11±1.75	12.39±1.05*
BGP	4.91±0.55	9.35±0.79*^△^	4.89±0.57	7.21±0.68*
β-CTX	0.98±0.21	0.58±0.11*^△^	1.05±0.23	0.75±0.12*

***Note:*** Compared with values before treatment of the same group,

* *P<*0.05; comparison with the control group after treatment,

△ *P<*0.05.

VAS and ODI scores in both groups after treatment are below those before treatment (*P*<0.05), while SF-36 scores after treatment are above those before treatment (*P*<0.05).Table-IV Moreover, VAS and ODI scores obtained from the treatment group after treatment are calculated to be below those of the control group, while SF-36 scores of the former are above those of the latter (*P*<0.05).

**Table- IV:** Comparison of VAS, ODI and SF-36 scores before/after treatment in observation and control groups (*χ̅*±*S*) Unit: Points.

Items	Treatment group (n=40)		Control group (n=40)	
	Before treatment	After treatment	Before treatment	After treatment
VAS score	6.40±0.74	0.83±0.38*^△^	6.35±0.74	1.75±0.44*
ODI score	40.08±6.38	12.30±1.42*^△^	41.28±6.33	20.08±1.53^*^
SF-36 score	53.38±5.53	87.96±5.67*^△^	51.98±5.60	73.20±4.41*

***Note:*** Compared with values before treatment of the same group,

* *P<*0.05; comparison with the control group after treatment,

△ *P<*0.05.

It is clear that the overall response rate of the treatment group (90.0%) is superior to that of the control group (67.5%) (*P*<0.05). Table-V. During treatment, one case of nausea and one case of dizziness were found in the treatment group, producing an adverse reaction occurrence rate of 5.0% (2/40). As far as the control group was concerned, one case showed nausea, one case showed dizziness and two cases were attacked by abdominal distension. In this event, the adverse reaction occurrence rate of the control group is worked out to be 10.0% (4/40). There is no statistically significant difference between the two groups (*P*>0.05).

**Table-V:** Clinical efficacy comparison between treatment and control groups [Case number (%)].

Groups	Case number	Significantly effective	Effective	Ineffective	Overall responses
Treatment group	40	10(25.0)	26(65.0)	4(10.0)	46(90.0)*
Control group	40	5(12.5)	22(55.0)	13(32.5)	27(67.5)

***Note:*** Through comparison with the control group,

* *P<*0.05.

## DISCUSSIONS

This retrospective study demonstrated that the combination of Teriparatide (TPTD) and Zhuanggu Zhitong Capsules (ZZC) for six months was associated with significantly greater improvements bone density, regulated bone metabolism, reduced pain and disability, and enhanced quality of life in patients with primary osteoporosis, with a higher total effective rate and comparable safety to Teriparatide monotherapy. Our findings regarding the superior efficacy of the combination therapy are consistent with the preliminary results from a previous smaller study, which suggested benefits of ZZC in postmenopausal osteoporosis.^8^ The magnitude of BMD improvement in our control group (TPTD alone) is aligned with expectations from international clinical trials and reviews.^9^,^10^ However, the significant additional benefit observed in the combination group suggests a potential synergistic effect between TPTD and the multi-component actions of ZZC.

The rationale for combining TPTD and ZZC is supported by their distinct yet complementary mechanisms. TPTD, a potent anabolic agent, directly stimulates osteoblast activity and new bone formation.^11^,^12^ According to Traditional Chinese Medicine (TCM) theory, primary osteoporosis is often attributed to kidney deficiency and blood stasis.^13^,^14^ ZZC, as a TCM formula, is designed to tonify the liver and kidney, invigorate blood circulation, strengthen bones, and relieve pain.^15^ Modern pharmacological studies have shown that key components of ZZC, such as Fructus Psoraleae and Herba Epimedii, can promote osteoblast proliferation, inhibit osteoclast activity, and regulate estrogen levels, which may contribute to its anti-osteoporotic effects.^16^,^17^ This multi-targeted approach of ZZC may enhance the bone-forming effects of TPTD and provide broader symptomatic relief, potentially explaining the superior outcomes in pain, function, and quality of life observed in our study.

### Strengths of the study:

Firstly, to our knowledge, it is one of the first retrospective cohort studies to directly compare the combination of TPTD and ZZC against TPTD monotherapy. Secondly, we employed a comprehensive set of outcome measures, including objective BMD and biochemical markers, as well as patient-reported outcomes for pain, function, and quality of life.

### Limitations:

Strengths include the retrospective cross-sectional design, which allowed for a relatively large sample size (n=80) and rigorous matching of baseline characteristics. However, limitations must be acknowledged: Secondly the study was retrospective, relying on EMRs, which may have introduced selection bias; we did not assess radiographic indicators (e.g., vertebral fractures, calcification of the coracoclavicular ligament) or patient-reported outcomes beyond standardized scales; Another limitation is that the follow-up period (6 months) was short, precluding evaluation of long-term efficacy or safety.

## CONCLUSIONS

The combination of Teriparatide and Zhuanggu Zhitong Capsules is a promising therapeutic option for primary osteoporosis, demonstrating superior efficacy in improving bone density, regulating bone metabolism, and reducing pain/disability compared to Teriparatide alone, with acceptable safety.

### Future prospective studies:

Ideally larger randomized controlled trials (RCTs) with longer follow-up periods, are warranted to confirm our findings. These studies should incorporate more detailed radiographic assessments and standardized monitoring of adverse events to further validate the synergistic effect and safety profile of this combination therapy.

### Authors’ Contributions:

**MC** and **PL:** Carried out the studies, participated in collecting data, drafted the manuscript, are responsible and accountable for the accuracy or integrity of the work. **SW:** Performed the statistical analysis, critical review and participated in its design. **XW, LY:** Participated in acquisition, analysis, or interpretation of data draft the manuscript.

All authors read and approved the final manuscript.
